# Acute bilateral retina hemorrhages beneath internal limiting membrane: An unusual ophthalmological case report of acute leukemia during complete clinical remission

**DOI:** 10.1097/MD.0000000000010000

**Published:** 2018-02-16

**Authors:** Shuyuan Lyu, Ming Zhang, Yunxia Gao

**Affiliations:** Department of Ophthalmology, West China Hospital of Sichuan University, Wuhou, Chengdu, Sichuan, China.

**Keywords:** leukemic retinopathy, lymphoblastic leukemia, retina hemorrhages beneath internal limiting membrane

## Abstract

**Rationale:**

Leukemia is a common hematologic disease that causes various systemic complications, such as ophthalmological disorders. The venous congestion is considered to be the main clinical sign that occurs during the initial stage of the disease, whereas white-centered hemorrhages are the most typical manifestations in leukemic retinopathy. These complications usually manifest when the disease presents with clinical and hematological symptoms. In the present study, we report a patient who was diagnosed with leukemic retinopathy. The initial signs of this disease were bilateral hemorrhages that occurred during complete clinical remission. Previous studies regarding this condition are quite rare.

**Patient concerns:**

We report a 26-year-old man who was diagnosed with leukemic retinopathy and exhibited the initial signs of the disease, namely bilateral hemorrhages with a distinct appearance beneath the internal limiting membrane. In addition, flame-shaped hemorrhages were observed surrounding the optic discs and/or along the vessels in the absence of venous congestion. All these changes were present during complete clinical remission.

**Diagnoses:**

Bilateral acute leukemic retinopathy, acute lymphoblastic leukemia (pro-B lymphocyte, BCR-ABL chimeric gene-positive).

**Interventions:**

Clinical remission was achieved following effective systemic chemotherapy that was applied for leukemia in the hematology department. A dynamic observation was applied actively in the absence of surgery and/or medical treatment for ophthalmologic treatment.

**Outcomes:**

Best corrected visual acuity was 20/40 in the right eye and 20/60 in the left eye, which was considerably better than those noted at the initial visit of the patient (20/250 in the right eye and 20/400 in the left eye).

**Lessons:**

The cautious expectant treatment is safe and helpful for acute leukemic retinopathy. A long-term follow-up is inevitable. Effective systemic chemotherapy that is required for leukemia treatment can achieve clinical remission, which might be helpful in controlling the pathological changes of the eyes.

## 1 Introduction

Leukemia is a common hematologic disease that causes various systemic complications, such as ophthalmological disorders. According to previous studies, both acute and chronic leukemia can cause leukemic retinopathy, although acute leukemia is the main cause of this condition.^[1,2]^ The intraocular manifestations of leukemia are common,^[1–8]^ whereas the intraocular manifestations of leukemic retinopathy are diverse. Previously reported findings that are associated with this conditions included venous congestion, retinal edema, hemorrhages, optic nerve infiltrates, and nonspecific uveitis.^[3–8]^ These complications usually occur when the disease presents with clinical and hematological symptoms, and are rare during complete remission.^[3,7]^ In the present study, we report a patient with bilateral intraretinal hemorrhages and acute lymphoblastic leukemia (pro-B lymphocyte, BCR-ABL chimeric gene-positive) in complete remission.

## 2 Case report

**Figure 1:**
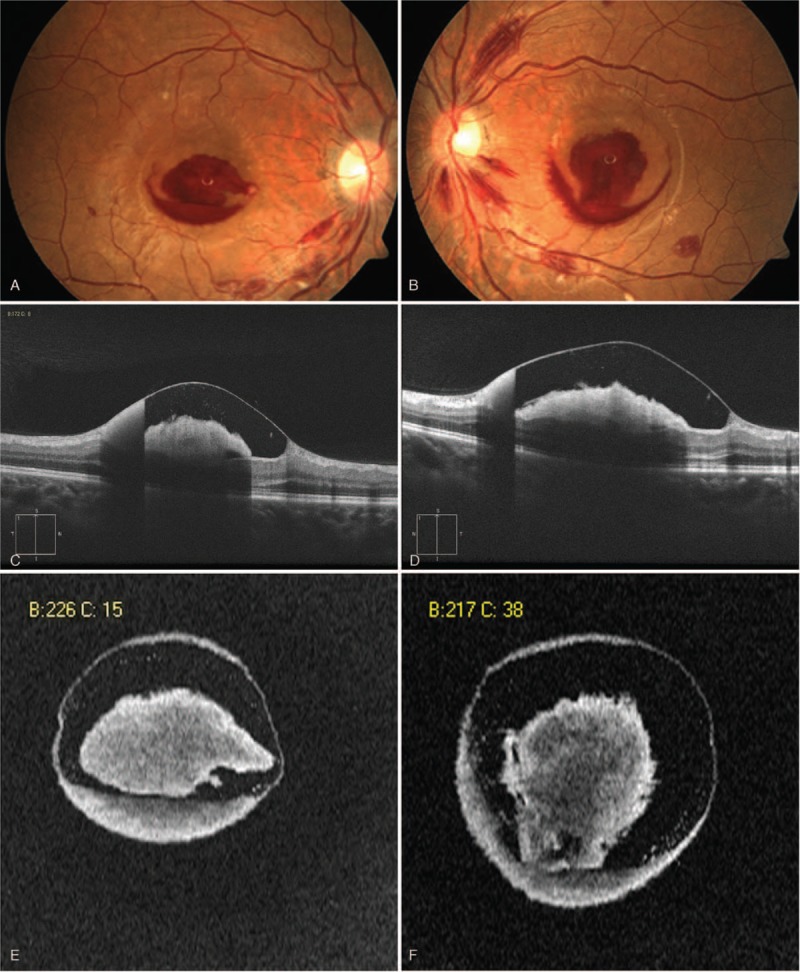
(A) Fundus color photograph of the right eye on first eye examination. The retina hemorrhages formed a special “sunset sign” on fundus color photographs. In addition, certain flame-shaped hemorrhages surrounding the optic discs or along the vessels were noted. (B) Fundus color photograph of the left eye on first eye examination, which resembled that noted on the right eye. (C) OCT B frame of the right eye on first eye examination. The shape of hemorrhages on cross-sectional OCT image of the right eye resembled a “rainbow” straddling the macular. The internal limiting membrane was detached, while the deep layers thickened. (D) OCT B frame of the left eye on first eye examination, which was similar to those noted in the right eye. (E) OCT Enface image of the right eye on first eye examination. Round lesions with thin wall, uneven continent, and liquid level were observed. (F) OCT Enface image of the left eye on first eye examination, which were similar to those noted in the right eye. OCT = optical coherence tomography.

A 26-year-old man with the main complaint of “bleeding gums, sore throat, petechiae, and melena” was diagnosed with acute lymphoblastic leukemia (pro-B lymphocyte, BCR-ABL chimeric gene-positive) by bone marrow biopsy, flow cytometry analysis of B cells, and genetic analysis, when marked leukocytosis (94.24 × 10^9^/L), thrombocytopenia (31 × 10^9^ /L), and anemia (103 g/L) were present. The proportion of leukemic blast cells to the peripheral blood cells was 85%. During the next 5 months, he received 4 cycles of standard chemotherapy. Complete clinical remission was achieved during the chemotherapy and was based on bone marrow biopsy and flow cytometry. A total of 13 days following the last chemotherapy cycle, he suddenly developed a blurred vision. During the first eye examination, his best corrected visual acuity (BCVA) was 20/250 in the right eye and 20/400 in the left eye, which was later attributed to an acute intraretinal hemorrhage that was present between the nerve fiber layer and the internal limiting membrane. This hemorrhage formed a special “sunset sign” on fundus color photographs (Fig. 1A and B) and “rainbow sign” on optical coherence tomography (OCT) B frames (Fig. 1C and D). Round lesions with thin wall, uneven continent, and liquid level were shown on Enface images (Fig. 1E and F). During diagnosis of the hemorrhage, the white blood cell and platelet counts were 3.60 × 10^9^ /L and 74 × 10^9^ /L, respectively, whereas the hemoglobin levels were 45 g/L.

A dynamic observation was applied actively without surgery and/or medicine for ophthalmologic treatment in the following months. The first follow-up eye examination was carried out 19 days later, and the second follow-up eye examination was 2 months later. BCVA, OCT, and fundus color photographs were also recorded during the 2 follow-up examinations. The institutional ethics review board policy was not required for the observational case report because it did not alter the patient management. Written informed consent was obtained from the patient for the publication of the findings.

## 3 Outcomes

**Figure 2:**
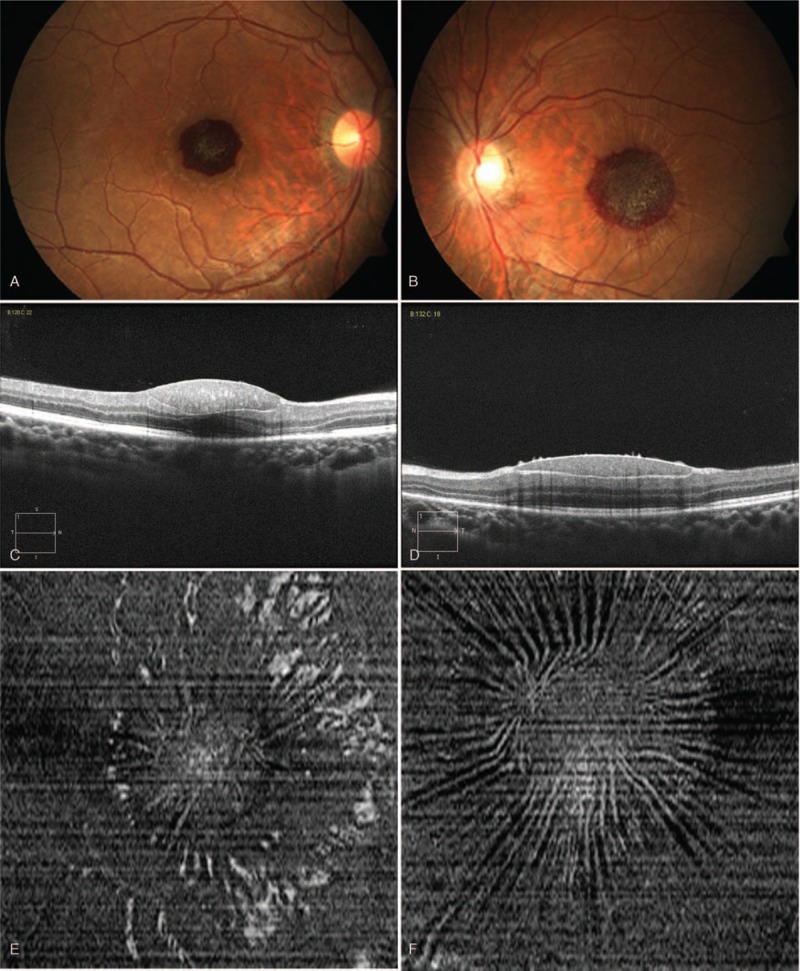
(A) Fundus color photograph of the right eye on the first follow-up examination. The liquid of the subretinal hemorrhage had been absorbed and the cellular components were localized at the left region, forming a deep red lesion involving the macula. (B) Fundus color photograph of the left eye on the first follow-up examination, which was similar to that noted for the right eye. (C) OCT B frame of the right eye on the first follow-up examination. Moderate-intensity spindle-shaped reflections were lying between the nerve fiber layer and the internal limiting membrane. (D) OCT B frame of the left eye on the first follow-up examination, which was similar to that noted for the right eye. (E). OCT Enface images of the right eye on the first follow-up examination. The internal limiting membrane was retracted and its appearance resembled a “sunburst” pattern. (F) OCT Enface images of the left eye on the first follow-up examination. The retraction of the internal limiting membrane was apparent compared with that noted in the right eye. OCT = optical coherence tomography.

**Figure 3:**
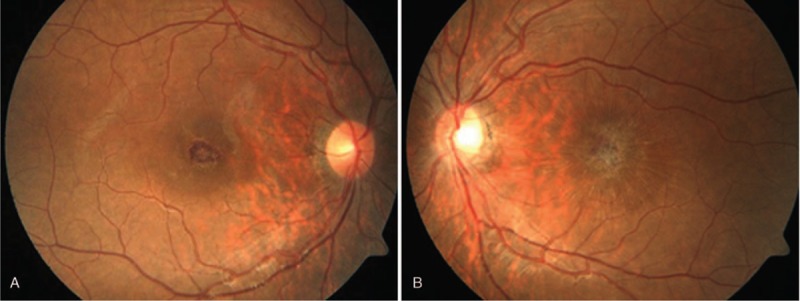
(A) Fundus color photograph of the right eye on the second follow-up examination. A small, dark red, submacular hemorrhage (about 1/2 papilla diameter in size) was noted. (B) Fundus color photograph of the left eye on the second follow-up examination. The hemorrhage of the left eye had disappeared, forming radial folds with traction toward the center.

On the first follow-up examination, BCVA parameters were very similar to those noted during diagnosis of the hemorrhage. OCT and fundus color photographs were carried out with the same scan settings in order to ensure comparability. The liquid of the subretinal hemorrhage was absorbed and the cellular components (Fig. 2A and B) formed a moderate-intensity spindle-shaped reflection lying between the nerve fiber layer and the internal limiting membrane as determined by OCT B frame images (Fig. 2C and D). The internal limiting membrane was retracted and formed a “sunburst sign” in Enface images (Fig. 2E and F). On the second follow-up examination, the BCVA parameters were 20/40 in the right eye and 20/60 in the left eye, respectively, which were considerably better than those noted at the initial visit of the patient (20/250 in the right eye and 20/400 in the left eye). The subretinal hemorrhage of the left eye was absorbed, while a small submacular hemorrhage (about 1/2 papilla diameter in size) was noted in the right eye (Fig. 3A and B).

## 4 Discussion

Leukemic retinopathy is mainly characterized by marked venous congestion, retinal edema, and white-centered hemorrhages.^[3–7]^ The retinal vessels are dilated, tortuous, and pale sheathed, due to the leukocytic infiltration of the vessel walls that produces a granular appearance of a blood column.^[2,3]^ The white-centered hemorrhages (“Roth spots”) are typical in leukemic retinopathy, which is formed by accumulations of white blood cells and fibrous exudation in the center of the hemorrhagic area.^[4]^ Previous studies have reported that all the pathophysiological changes that have been associated with leukemic retinopathy may present with high viscosity, leukocytosis, thrombocytopenia, and severe anemia.^[5,7]^

The present case report exhibits several distinctive clinical features. First, retina hemorrhage occurred during complete clinical remission, when the hematocrit (HCT) and the hemoglobin levels were 0.14 L/L (normal: 0.40–0.50 L/L) and 45 g/L (normal: 120–160 g/L), respectively. In addition, these changes were observed in the presence of slight thrombocytopenia and leucopenia. This suggested that retinal hemorrhage was closely associated with anemia, as determined by the decreased HCT and hemoglobin. It has been proposed that anemia plays an important role in leukemic retinal hemorrhage, notably in the decrease of HCT, which can cause retinal hemorrhage even during complete clinical remission. Second, the initial changes of fundus that were noted in the present case report, were bilateral hemorrhages beneath the internal limiting membrane and flame-shaped hemorrhages surrounding the optic discs and/or along the vessels. Previous studies regarding this condition are limited. The venous congestion is considered to be the main initial sign of leukemic retinopathy^[2,7,8]^ and the development of Roth spots is the most typical manifestation related to leukocytosis that is derived from leukemic retinopathy. Both of these symptoms exhibit a close relationship with leukocytosis. In the present case, Roth spots and venous congestion were not observed. We believe that this was due to the high level of the white blood cells of the patient who did not last long enough in order to cause pathological changes owing to his optimal response to systemic chemotherapy. The pathological changes of fundus in the early stage of the present case report were most likely caused by severe anemia. Furthermore, the intraretinal hemorrhage produced a characteristic “sunset sign” that was determined by fundus color imaging and a “rainbow sign” that was determined by OCT B frame. Moreover, uneven continent and liquid levels, frames, and round lesions with the thin wall were reported by Enface imaging. The massive intraretinal hemorrhage caused a sudden detachment of the internal limiting membrane, which in turn damaged the deep layer. The structures beneath the internal limiting membrane were expanded toward the vitreous cavity to form a nearly round mound. The sedimentation of the blood allowed the distinction of the hemorrhage by a characteristic line demarcating the superior transparent plasma from the inferior corpuscular part of the hematocele. The lower part of the mound shape structures beneath the internal membrane was internalized by the inferior corpuscular part of the hematocele, while the higher part was noted via the transparent plasma, forming a distinctive “sunset sign” on fundus color imaging. The shape of this structure on the cross-sectional OCT image resembled that of a “rainbow” straddling the macular.

## 5 Conclusions

Following a period of dynamic observation at the last follow-up examination, the BCVA of the patient improved (20/40 in the right eye and 20/60 in the left eye) compared with that noted during his initial visit (20/250 in the right eye and 20/400 in the left eye). This indicated that cautious expectant treatment is safe and helpful for this type of acute leukemic retinopathy. We suggest that the principles for expectant treatment are as follows: the risk/benefit ratio should be evaluated prior to the therapeutic decisions. Because patients with hematological disorders exerted a much higher risk of bleeding and infection than the normal subjects, noninvasive treatment strategy, such as the expectant ophthalmic treatment should be the first choice of treatment. The proper surgical treatment should be performed in case of active bleeding, vitreous hemorrhage, nonperfusion area, and/or neovascularization. A cautious strategy should be maintained and the purpose of surgery should be to preserve the existing vision and avoid serious complications rather than to eradicate the pathological changes. A long-term follow-up should be conducted in order to monitor the progression of the disease, because the pathogenic condition of leukemia is complicated and dynamic. Effective systemic chemotherapy for the treatment of leukemia can achieve clinical remission, which might be helpful in controlling the pathological changes of the eyes.
